# Supplementary material for: Molecular dynamics shows complex interplay and long-range effects of post-translational modifications in yeast protein interactions
Source: PLoS Comput Biol. 2021 May 12;17(5):e1008988. doi: 10.1371/journal.pcbi.1008988 (PMC8143416; doi:10.1371/journal.pcbi.1008988)
Supplement: S1 README — (PDF) [file pcbi.1008988.s004.pdf]

Sostaric, Nikolina, & van Noort, Vera. (2020). Molecular dynamics shows complex interplay and long-range effects of post-translational modifications in yeast protein interactions (Version v3) [Data set]. Zenodo. http://doi.org/10.5281/zenodo.4099098

The data set contains following files:

1. Input coordinates
Coordinates files for all protein complexes after parametrization in teLeap, used for optimization and subsequent MD steps.
Files are named as [PDB_ID]_[PTM_status]_tleapout.crd, where PTM_status can be noPTM (non-modified complex), NC_PTM (complex modified with PTMs found in Normal Conditions), or SC_PTM (complex modified with PTMs found in Stress Conditions).

2. Input topology
Topology files for all protein complexes after parametrization in teLeap, used for optimization and subsequent MD steps. Naming scheme is analogous to 1. ([PDB_ID]_[PTM_status]_tleapout.top).

3. Input structures
PDB files corresponding to protein complexes in explicit water. Naming scheme is analogous to 1. ([PDB_ID]_[PTM_status]_tleapout.pdb).

4. Scripts and parameters

> Optimization and equilibration
- Optimization_Equilibration.pbs (script for running 5 cycles of optimization and 500 ps of equilibration with Amber using VSC infrastructure)
- min1-5.in (parameters files for each of the 5 minimization cycles)
- md0to300ps.in (parameters file for first 300 ps equilibration)
- md300to500ps.in (parameters file for the second part of equilibration, NpT ensemble)

> Production phase and analysis
- ParmEd_Amb2Gmx.py (transferring relevant files from Amber to Gromacs format)
- Production_phase.pbs (script for running 19.5 ns of MD production phase in Gromacs using VSC infrastructure)
- NPT_2fs.mdp (parameters file for MD production phase)
- MMGBSA_script.pbs (script for running MMGBSA calculation using VSC infrastructure, the example script is for calculating interaction between chain A and the remaining chains of the complex in a SYSTEM)
- MMGBSA_decomp.in (parameter files for running MMGBSA)

5. Trajectory snapshots
Trajectory files containing structural snapshots used for binding energy calculations (protein complexes stripped of solvent). The snapshots are provided instead of raw trajectory files because the total size of all 440 raw trajectories reaches approx. 3.7 TB, making deposition impractical. The authors will however readily share individual raw files with interested researchers.
The files are named as [PDB_ID]_[PTM_status]_10-20ns_100snap.trr, and are

dispersed among five folders in the repository based on the first character in the respective PDB ID, as indicated in folder names.

6. Topology stripped
Topology files corresponding to protein complexes stripped of solvent, which can be used to view the trajectory snapshots.
The files are named as [PDB_ID]_[PTM_status]_complex.top.

7. RMSD files
RMSD calculations for either backbones or entire protein complexes throughout the MD production phase.

8. Cluster representatives
PDB files for the representative structures of the largest cluster from 100 conformational snapshots taken from the last 10 ns of each simulation.

9. PTMs_map
Python script for automated addition of PTMs to protein structures (PDB) using PyTMs plugin in PyMOL. The script is given together with an example.
