# Supplementary figures and images for: Molecular dynamics shows complex interplay and long-range effects of post-translational modifications in yeast protein interactions
Source: PLoS Comput Biol. 2021 May 12;17(5):e1008988. doi: 10.1371/journal.pcbi.1008988 (PMC8143416; doi:10.1371/journal.pcbi.1008988)

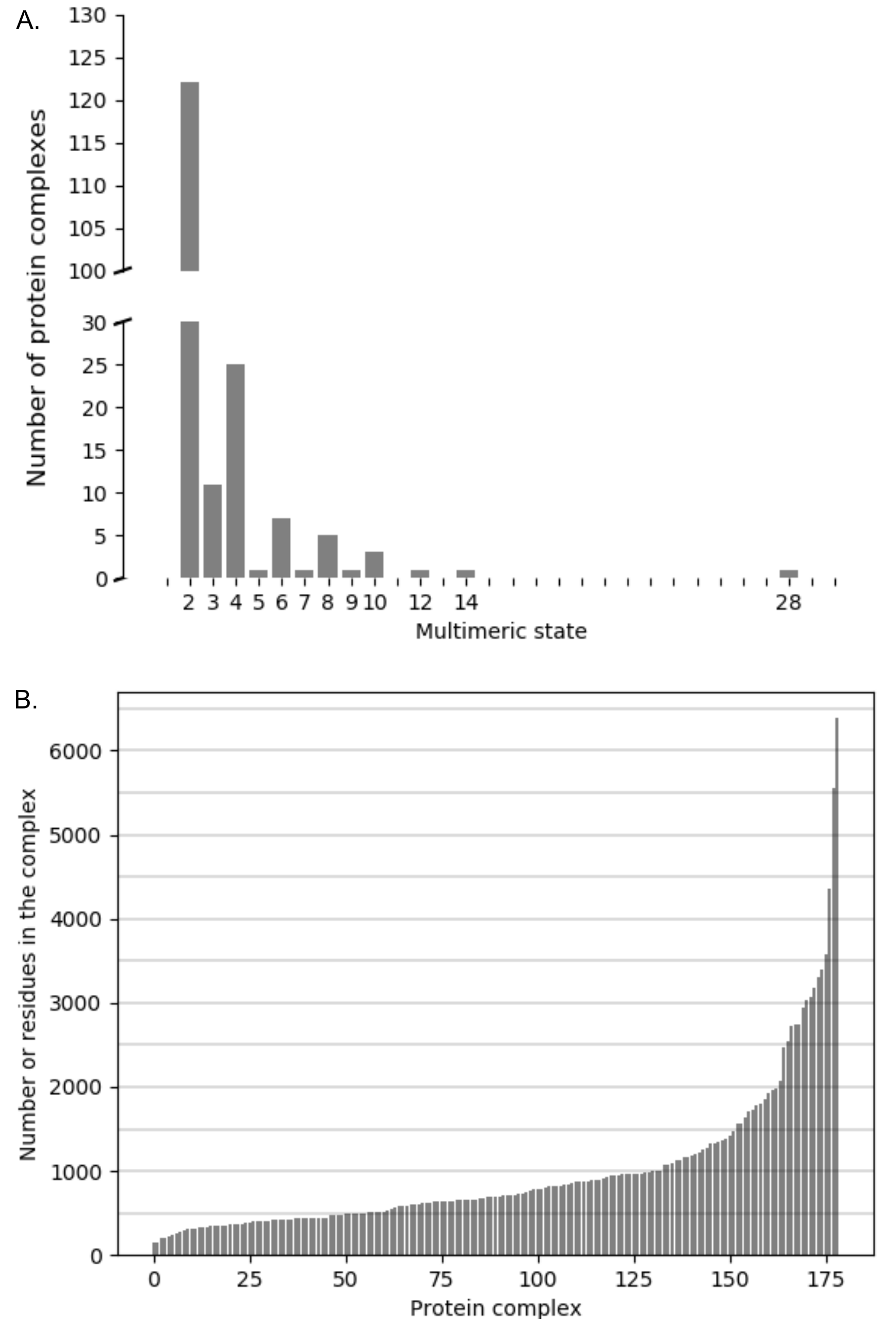

Supplement: S1 Fig — (TIF) [file pcbi.1008988.s005.tif]

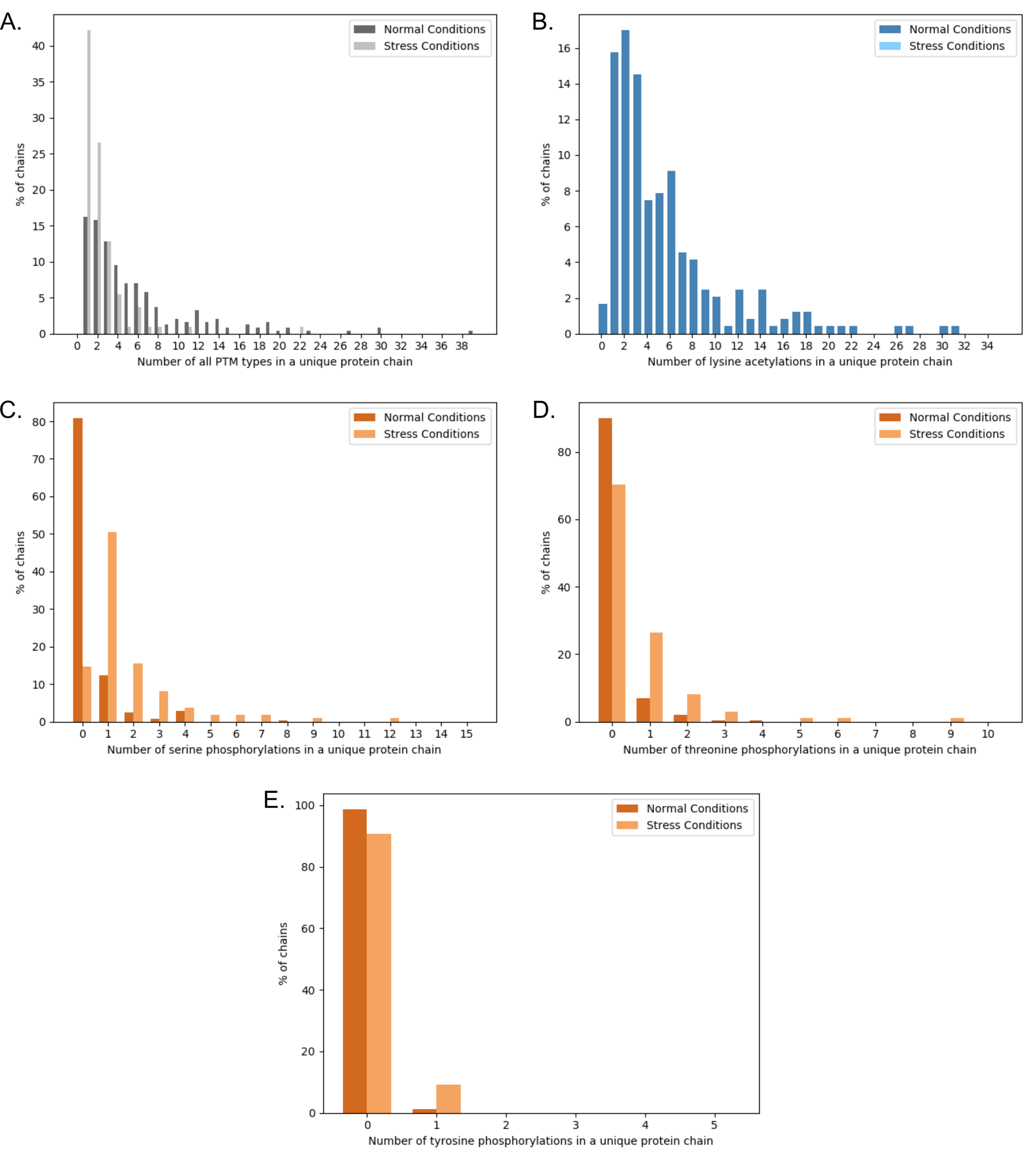

Supplement: S2 Fig — Distribution of the number of A. all PTM sites, B. lysine acetylation, C. serine phosphorylation, D. threonine phosphorylation, and E. tyrosine phosphorylation sites among unique protein chains in the analyzed dataset. The darker shade of color in each subplot denotes normal and lighter stress conditions. (TIF) [file pcbi.1008988.s006.tif]

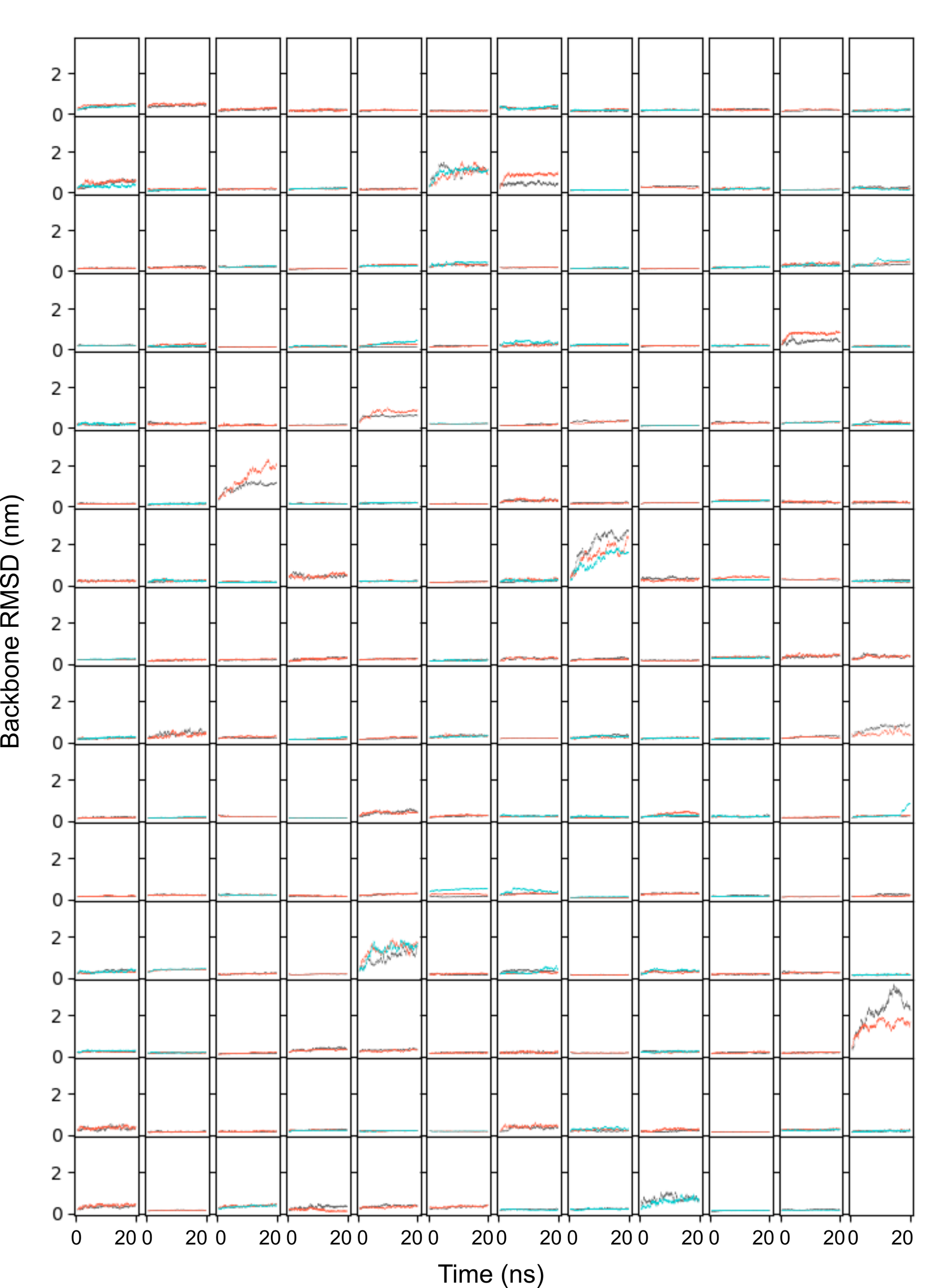

Supplement: S3 Fig — Grey denotes non-modified complexes, red complexes with PTMs in normal conditions, and cyan with PTMs in stress conditions. While most complexes are rather stable, the notable exceptions belong to the following systems: 1VLU, 2EKE, 4DL0, and 4WXA. (TIF) [file pcbi.1008988.s007.tif]

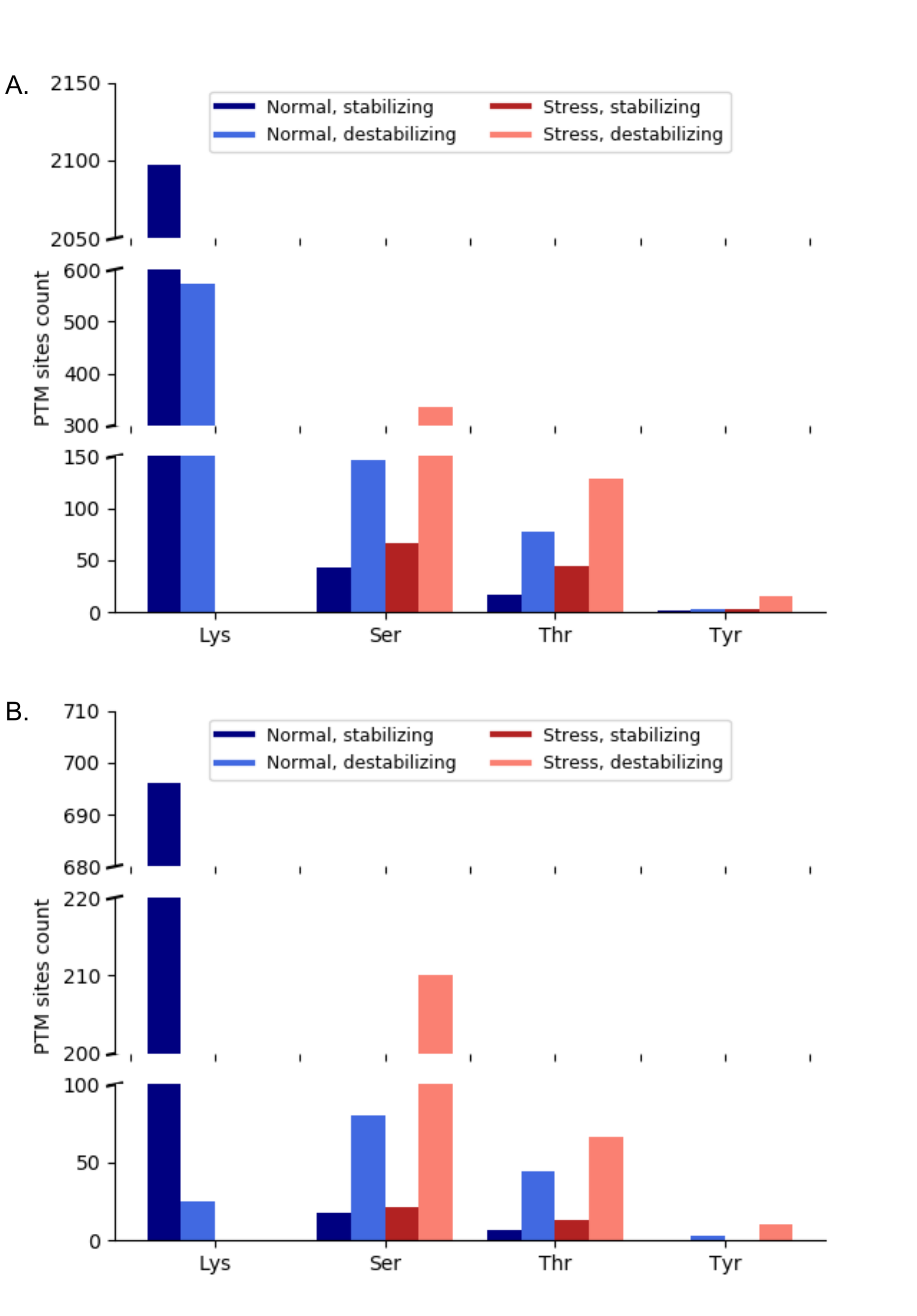

Supplement: S4 Fig — Local contributions of A. all PTMs and B. interface located sites (i.e., contributions above 0.5 kcal/mol or below -0.5 kcal/mol) to the overall binding (ΔΔGbind). (TIF) [file pcbi.1008988.s008.tif]

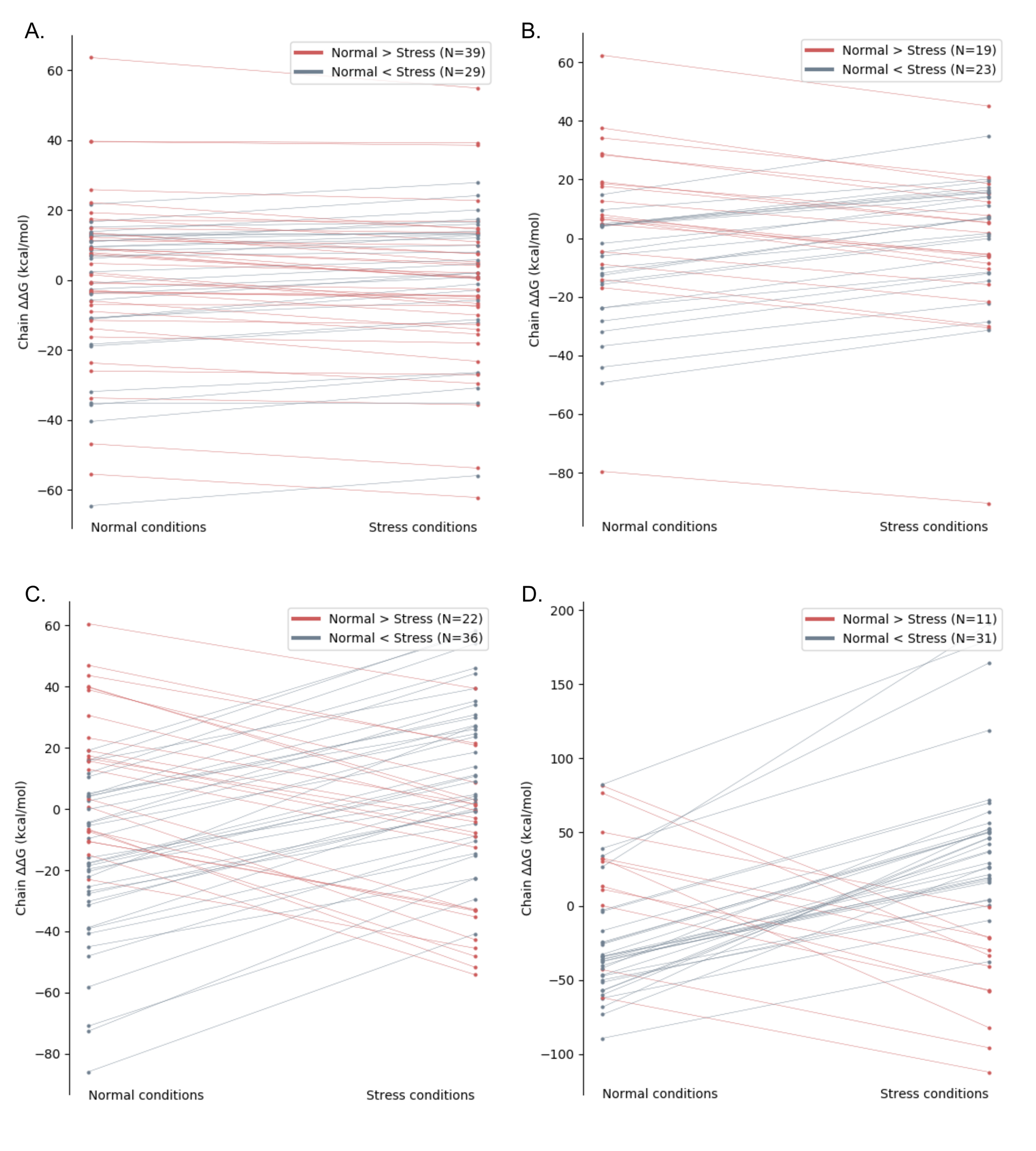

Supplement: S5 Fig — Lines connect the subunit ΔΔGbind values, where the color of the line denotes their relationship. For the sake of clarity, the data is split based on |ΔΔGbind,NC−ΔΔGbind,SC|: A. <10 kcal/mol, B. 10–20 kcal/mol, C. 20–50 kcal/mol, and D. >50 kcal/mol. (TIF) [file pcbi.1008988.s009.tif]

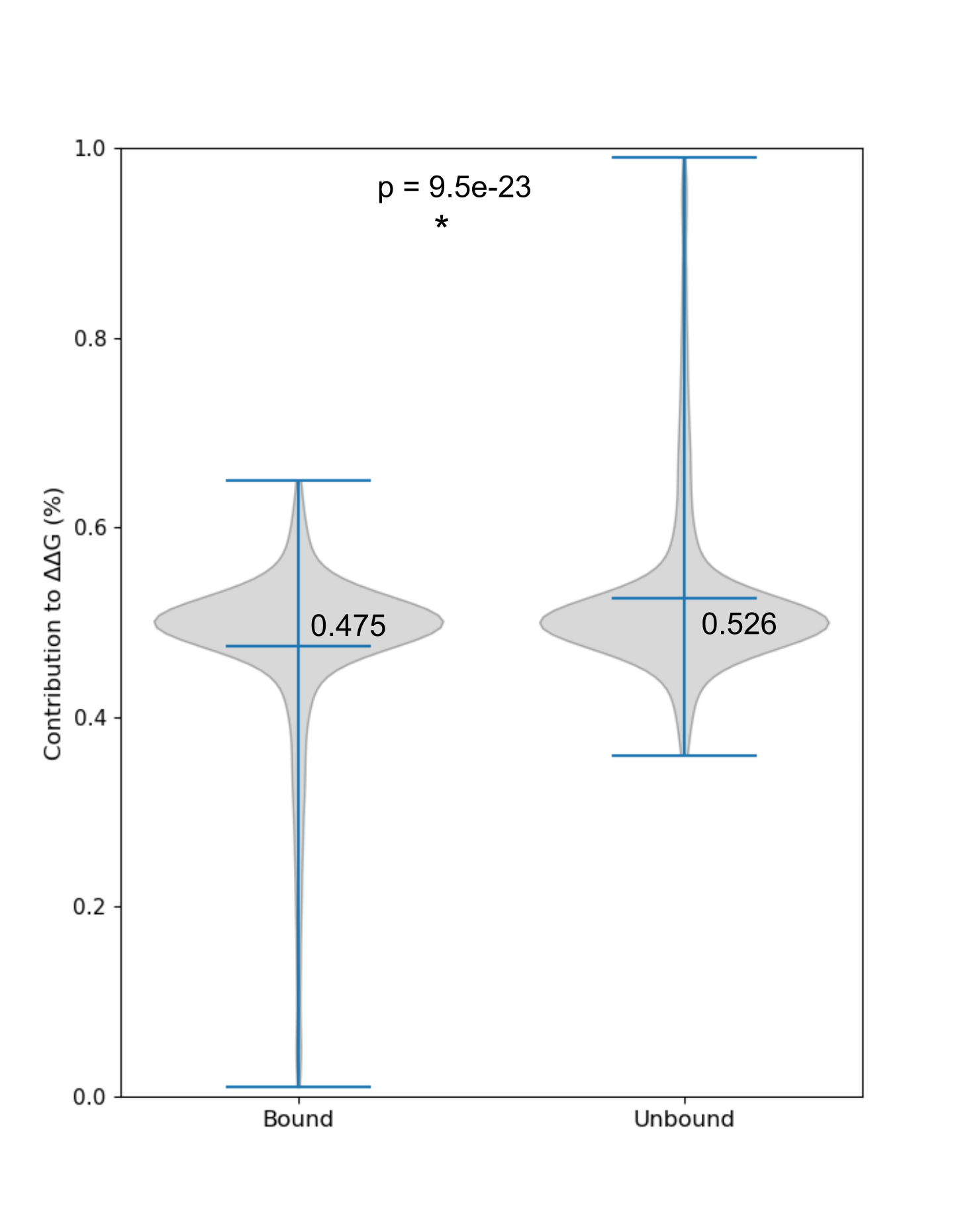

Supplement: S6 Fig — (TIF) [file pcbi.1008988.s010.tif]

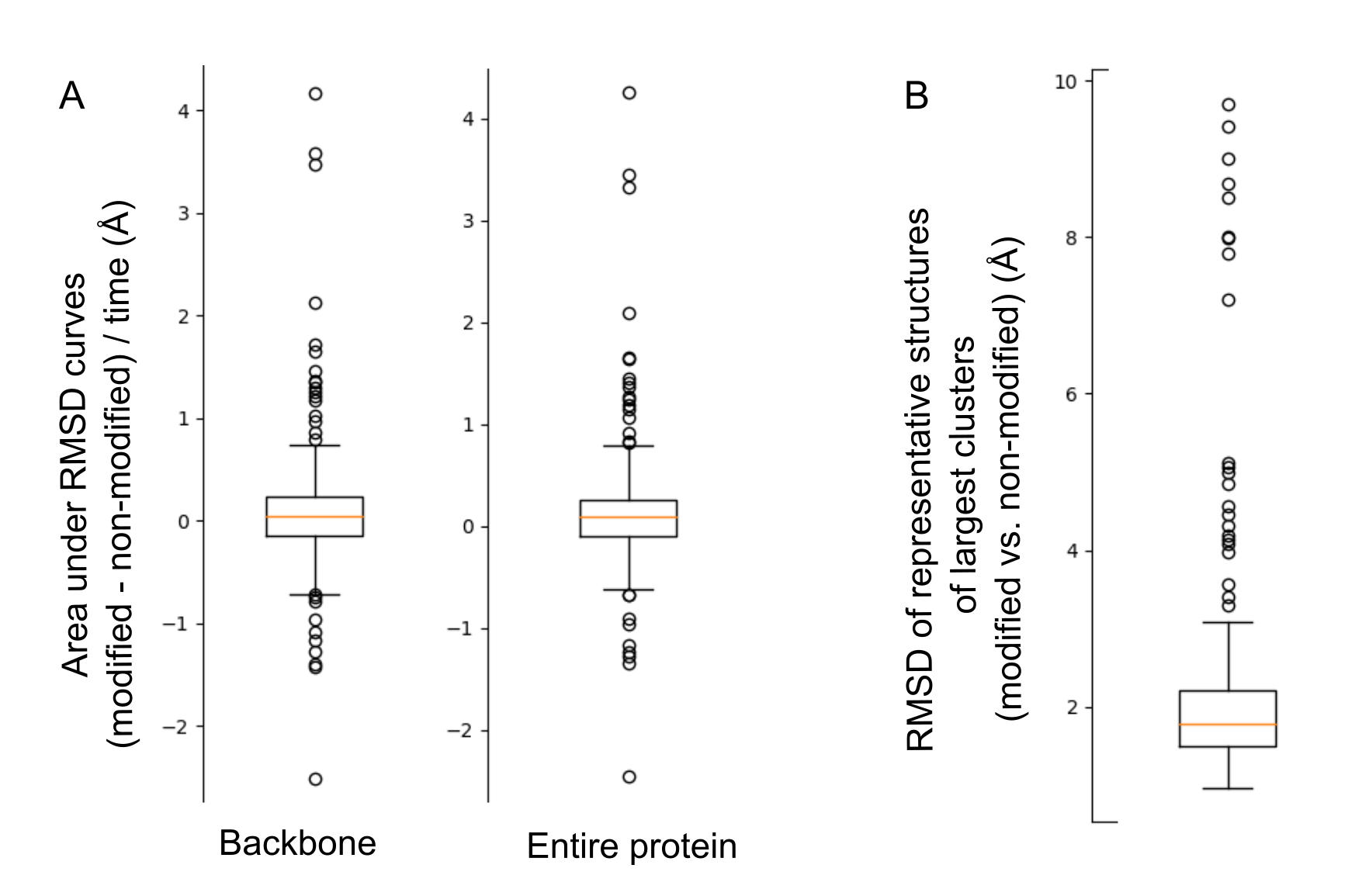

Supplement: S7 Fig — The difference of conformational changes in modified and non-modified protein complexes A. with initial structures used as reference points and B. when comparing representative structures of the largest conformational clusters in the final parts of trajectories. Data points for systems 1VLU, 2EKE, 4DL0, and 4WXA are excluded from the plots. (TIF) [file pcbi.1008988.s011.tif]

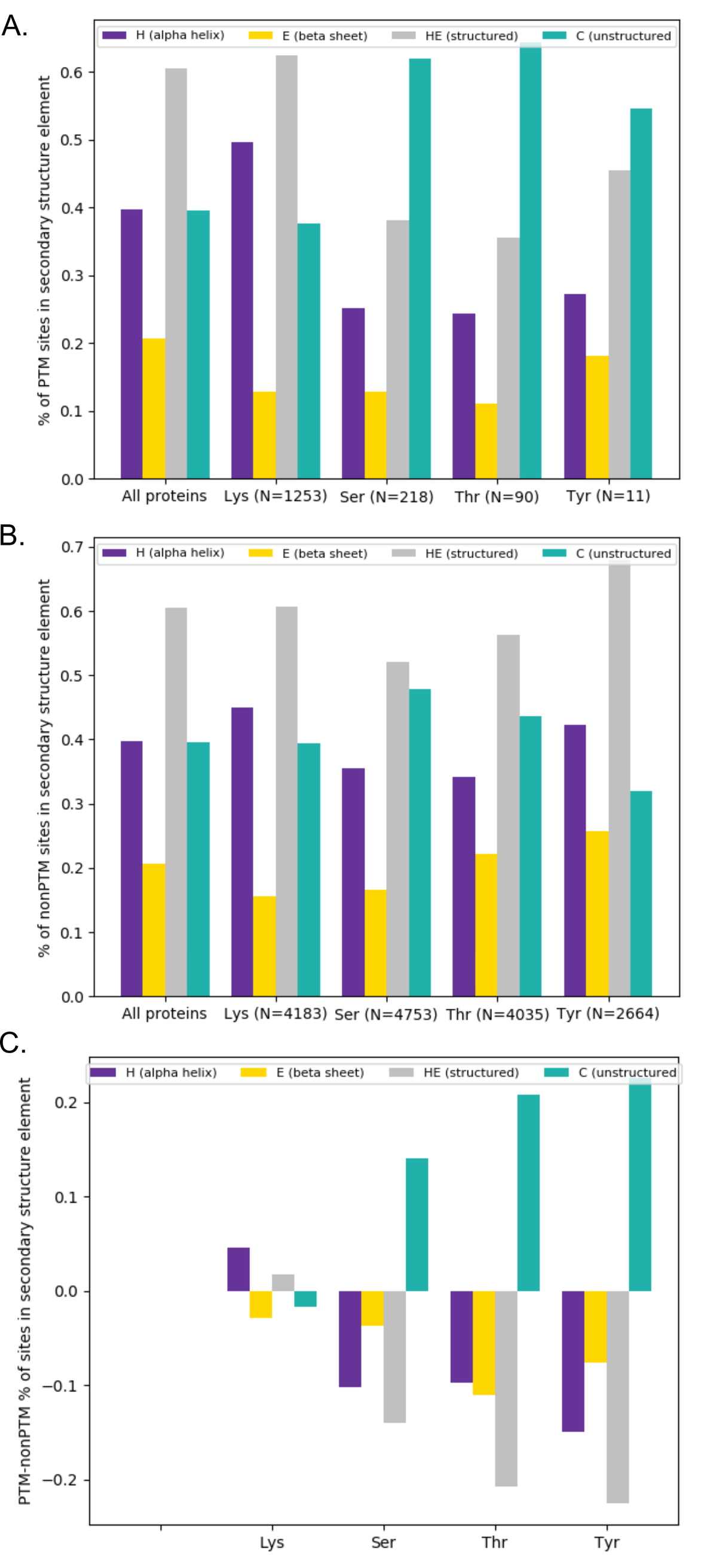

Supplement: S8 Fig — Amino acids placement within secondary structure elements in protein structures. Distribution of A. PTM sites and B. non-modified amino acids of the given type is shown, as well as C. their difference. “All proteins” bars describe the distribution of all amino acids in the secondary structure elements for the entire protein dataset, and serve as a reference. (TIF) [file pcbi.1008988.s012.tif]

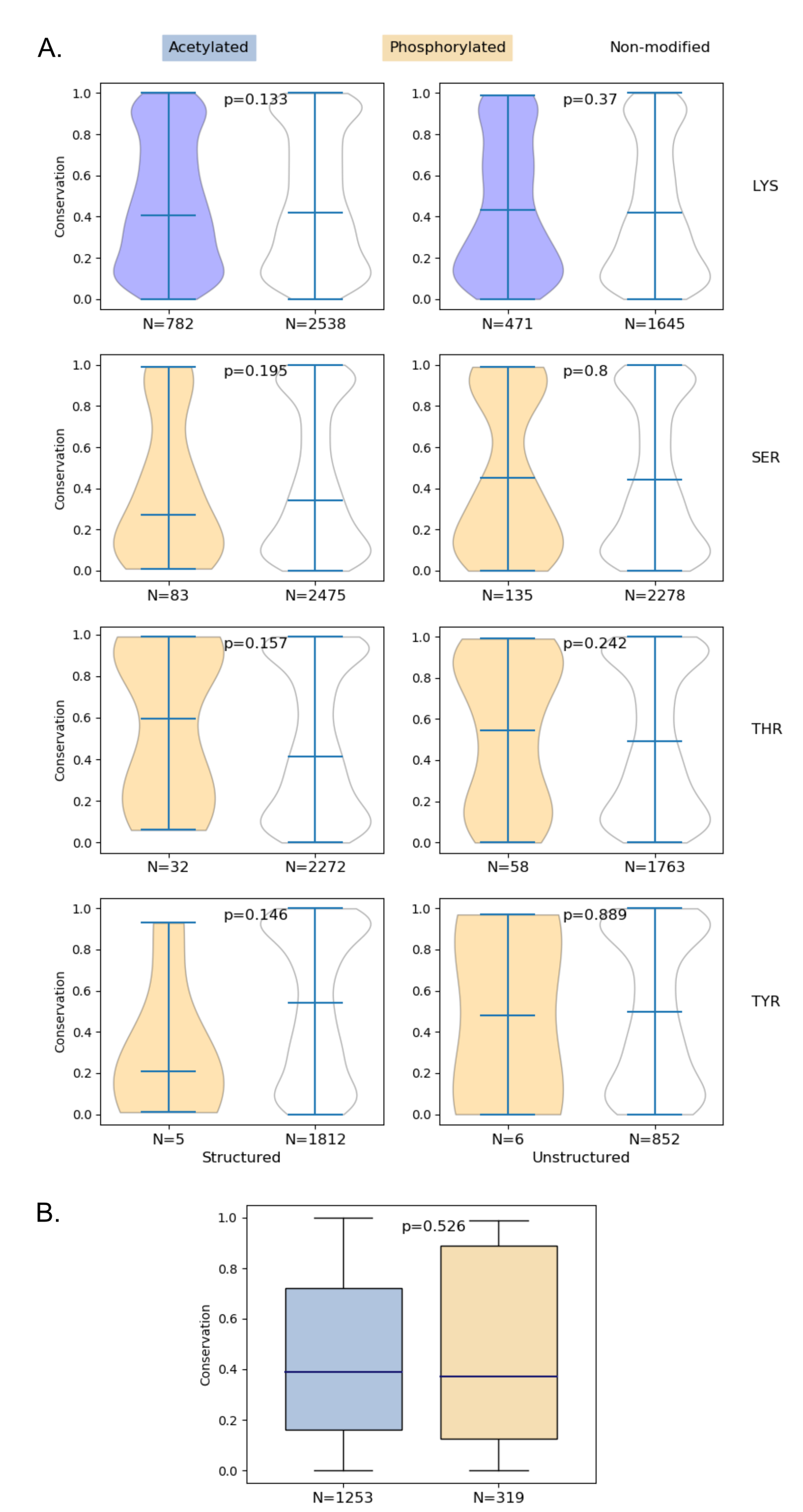

Supplement: S9 Fig — Comparison of the conservation levels. A. Even with the secondary structure taken into account, the PTM sites do not appear to be more conserved than the equivalent non-modified amino acids. B. No significant difference is found in the conservation of acetylation and phosphorylation sites. (TIF) [file pcbi.1008988.s013.tif]

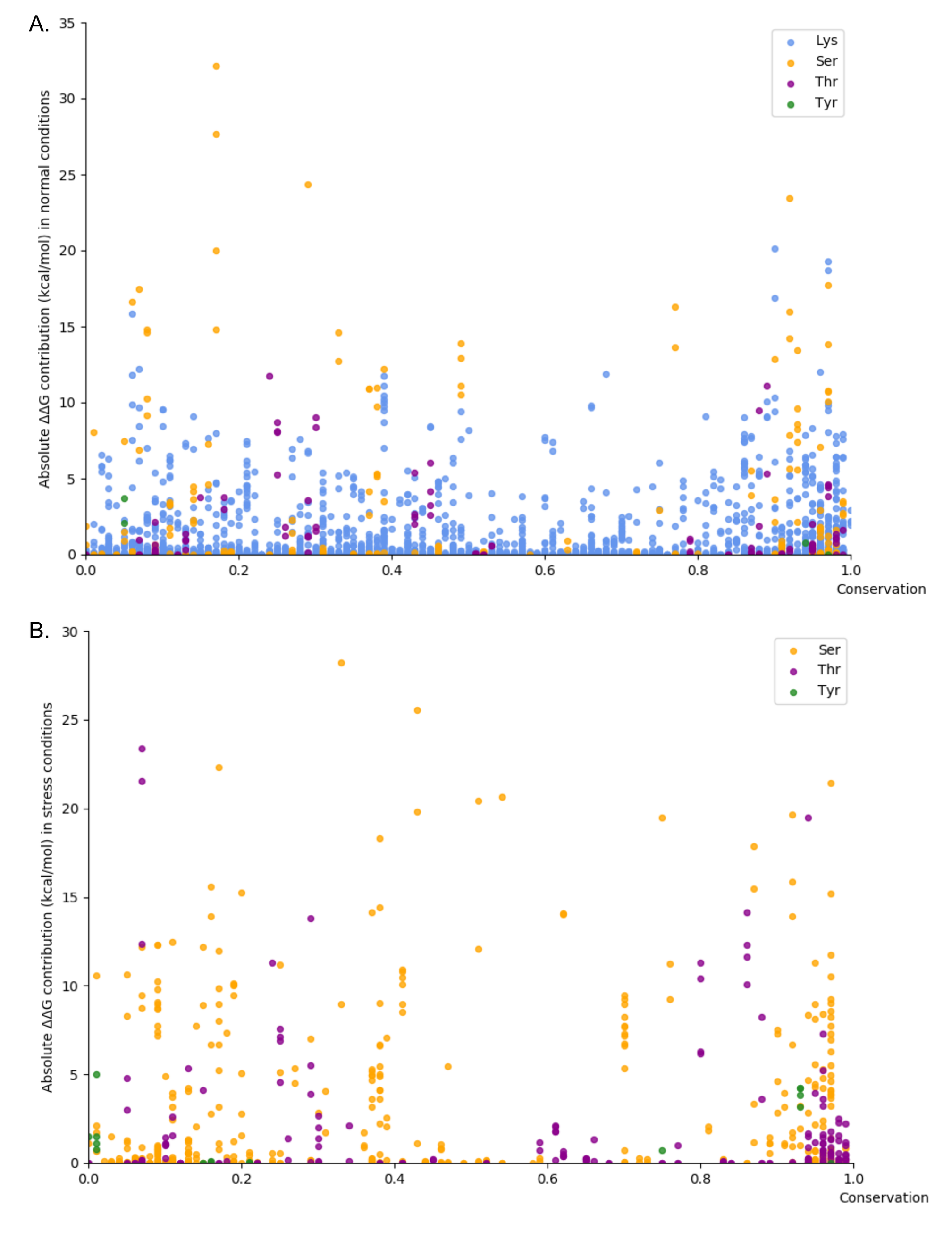

Supplement: S10 Fig — No correlation is observed in either A. normal or B. stress conditions for any of the PTM types (different colors), however, it is possible that PTMs with small contributions still do affect binding through long-range conformational changes, which is not captured by ΔΔGbind,contribution. (TIF) [file pcbi.1008988.s014.tif]

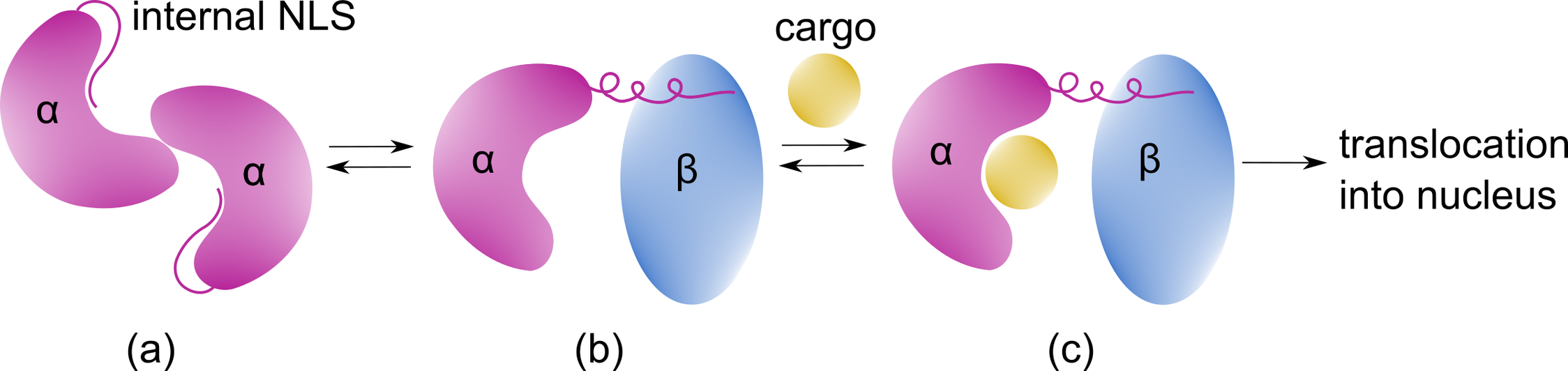

Supplement: S11 Fig — The role and regulation of importin alpha. (a) The auto-inhibition of importin alpha (α) was suggested to occur both by binding of the internal nuclear localization signal (NLS), as well as homodimerization. (b) Binding of importin beta (β) to importin alpha releases the auto-inhibition by disrupting the homodimerization and displacing the internal NLS. (c) Formation of the α:β heterodimer enhances recognition of NLSs in the cytosolic cargo proteins and their subsequent translocation to the nucleus. Based on Goldfarb et al. (2004) [28] Trends Cell Biol. and Conti et al. (1998) [29] Cell. (TIF) [file pcbi.1008988.s015.tif]
