# Supplementary material for: Molecular dynamics shows complex interplay and long-range effects of post-translational modifications in yeast protein interactions
Source: PLoS Comput Biol. 2021 May 12;17(5):e1008988. doi: 10.1371/journal.pcbi.1008988 (PMC8143416; doi:10.1371/journal.pcbi.1008988)
Supplement: Attachment — Submitted filename: review_PCOMPBIOL_2020.pdf [file pcbi.1008988.s016.pdf]

Review: Molecular dynamics shows complex interplay and long-range effects of post-translational modifications in yeast protein interactions

In this manuscript, the authors study the effect of acetylation and phosphorylation on the stability of protein complexes. Whereas acetylation appears to have a stabilizing effect, phosphorylation seems to destabilize interfaces. The impact of these post-translational modifications (PTMs) on the conformation of complexes was also studied by calculating the root-mean-square deviation of the complexes. These results were obtained by performing molecular dynamics simulations and free energy calculations on a filtered dataset and comparing trajectories of modified versus non-modified PTM systems.

General

Overall I think the manuscript shows interesting effects of the presence of PTMs and how multiple PTMs in one system can have a very different effect than individual PTMs in the same system. My main concern is the setup of the molecular dynamics simulations. Especially, since the padding of the systems was 10 Å of water molecules and the cutoff for non-bonded interactions was 12Å for the production phase. Because compression of the initial systems as well as conformational changes of the studied proteins is common in MD, there is a possibility that the simulated proteins reach a distance shorter than 12Å between the original protein in the unit cell and one of its periodic images. This should be carefully checked and if this artifact is found in one of the systems, these simulations should be discarded and rerun as this can affect the stability and conformation of the studied complexes. Another potential issue with the simulations could be the use of a Berendsen type thermostat for the production phase instead of a Nose-Hoover thermostat and the length of the produced trajectories.

Major

Section Post-translational modifications of yeast proteins. From line 143 to line 155, percentages of PTMs are described for different conditions. For reader clarity, would it be possible to translate these percentages into pie charts, for example, so the reader can get an overview of the discussed data in a figure as well?

Section Protein conformational changes due to PTMs. I think it would be good to also show the plain RMSDs for modified and non-modified systems in a supp figure to show that the RMSDs have converged in the production phase. The backbone RMSD will show the clear change in conformation. The RMSD of the entire protein with side chains can cloud the difference in conformations.

Section A case study – importin alpha. I think it would be helpful for clarity if the Importin mechanism of dimerization and activation could be illustrated with a figure in the supporting information for the first paragraph of this section.

Section Molecular dynamics simulations.
I would strongly advice to change the order of the simulation details. Especially the equilibration and production protocol descriptions are challenging to follow.

Line 475. It would be advantageous to repeat the description of the full protocol of protein structure preparation and MD used in this manuscript as well because a large part of the findings are based on MD data.
Line 487. Besides neutralizing the systems, was 150mM of salt also added to the systems?
Line 487, 494, 502. Cutoff for non-bonded interactions seems quite long (15Å equilibration, 12Å production) with respect to the water padding of 10 Å that was used. A major concern would be the protein in the unit cell “feeling” its periodic image. Authors should check for all trajectories if the original proteins and their periodic images are further apart than 12Å in the production runs. Some systems show large RMSDs, this could be caused by this artifact. It would be essential to check this and if the case, discard all systems with this periodic image issue.
Line 498. Why is there a switch from amber to gromacs? 19.5 ns of production is on the short side for letting the system converge and being able to check protein stability. Would it be possible to extend the simulations to 50 ns? Were replicates with different initial velocities run to verify the reproducibility of the MD simulations?
Line 500. The modified Berendsen thermostat is used. is this the V-rescale thermostat? Is there a reason why a Nose-Hoover thermostat was not used during production? According to my experience, Nose-Hoover is recommended as thermostat for production runs, V-rescale is usually used for the equilibration phase.
Line 501. Was an isotropic barostat used as all simulated systems were solvated?

Minor
Line 106. “that a significant proportions of proteome carries”. Proportions should be changed to proportion.

Line 109. “over-performs”, do the authors mean outperforms?

Lines 157-159. This sentence is a bit on the long side. Could this sentence be restructured?

Line 210. “PBD” should be changed to PDB.

Line 261-261. Can the authors also show the same trend for PTMs when you only take into account residues that are located at the protein-protein interface?

Line 348-349. Are the serines more often located at protein-protein interfaces than the threonines? Could this be a reason why serines have more impact on the stability than threonines?

Section Selection of protein structures. In the dataset, were only dimers considered or also larger complexes? It would be nice to know the distribution.

Figure 2. In order to increase the readability of the figure, maybe make clear in the figure which graphs concern phosphorylation and which ones acetylation. Using the same range for the y-axes would also make it easier to compare the different PTMs. It

could also be useful to put the numbers of the means in the graph for B and maybe also for A. It will make it easier for the reader to compare results.

Figure 4. It could have something to do with the PDF, but which plot is stress and which plot is normal conditions? Would it be helpful to print the means here as well?

Figure 5. How many PTMs were considered in total? How many sequences were taken into account per bar in the histogram, are the results normalized somehow per PTM? I am just wondering how one can compare the different bars in the histogram. To me it is unclear what these bars are composed of.

Figure 6. In the modified structures, I am assuming Lys463 and Ser163 are not acetylated and phosphorylated, respectively? If these residues are not modified, which nearest residues are modified and can the orientation with respect to the highlighted site be shown as well?

Supp Figure 1. It is a bit challenging to read. Could adding an additional representation help? For example, by clustering the runs by specific range in $\Delta\Delta G$ difference?

Supp Figure 3. Maybe also show the difference between A and B so it becomes clear very quickly what the change in the distribution of secondary structure elements is for the modified residues.
