# Supplementary material for: Molecular dynamics shows complex interplay and long-range effects of post-translational modifications in yeast protein interactions
Source: PLoS Comput Biol. 2021 May 12;17(5):e1008988. doi: 10.1371/journal.pcbi.1008988 (PMC8143416; doi:10.1371/journal.pcbi.1008988)
Supplement: Attachment — Submitted filename: Response_to_reviewers.pdf [file pcbi.1008988.s017.pdf]

Manuscript resubmission

Molecular dynamics shows complex interplay and long-range effects of post-translational modifications in yeast protein interactions

PCOMPBIOL-D-20-01903

Dear Dr. Dunbrack,
Dear Dr. Elofsson,

Thank you for the opportunity to resubmit our manuscript for publication as Research Article in PLOS Computational Biology. We appreciate the detailed review and constructive suggestions that we were provided with, and believe that our manuscript is significantly improved by the suggested edits.

Following this letter is our point-by-point response to the issues raised by the reviewers, including the description of changes made in the manuscript and the supplemental material.

For the authors,

Nikolina Šoštarić

Reviewer #1

The authors use equilibration molecular dynamics (MD) simulations, combined by the free energy calculation using MM/GBSA method to study the impact of phosphorylation and acetylation on the structure, dynamics and thermodynamics of protein complexes. The authors demonstrate find that acetylation tends to have locally stabilizing roles, while the opposite is seen in the case for phosphorylation. Importantly, the authors show that the modifications away from the binding site may significantly contribute to binding due to their effect on protein's structure. Given their ubiquity and biological importance, post-translational modifications are an extremely important and timely area of research and I find the authors' contribution to be relevant and publishable in PLOS Computational Biology, provided the following comments have been adequately addressed.

Major comments

1. The authors find that the acetylation in general acts in a stabilizing fashion, while phosphorylation is destabilizing. It is, however, not clear whether this effect is due primarily to the changes in the bound state, the unbound state or both. The authors should find a way to quantitatively estimate these contributions separately.

We thank the reviewer for this valuable suggestion. We further analyzed the MM/GBSA outputs and have made a quantitative estimation of the contributions of the bound and unbound state to the overall ΔΔG effects observed upon PTMs addition, starting from free energy values for complex, receptor and ligand obtained during each MM/GBSA calculation. Briefly, we find that whether the effect is primarily due to bound or unbound state varies from one protein system to the next. Overall, a bigger contribution is observed for the unbound than for the bound state (p = 5.8e-23; Cohen's d = 0.55, suggesting a medium size effect). One potential caveat (also emphasized in the Results of the revised manuscript) is the fact that the unbound states are inferred from the bound ones, given that only the structures of complexes, but not individual protein components, were subjected to MD before MM/GBSA calculations.

We have added 1. the detailed description of the procedure in "Binding energy calculation" section of the Methods, 2. description of the results as the second paragraph in "Co-occurring PTMs together affect protein interactions" section, 3. calculated values in the Supplemental Table S2 (sheet 3), and 4. a figure summarizing these results as Supplemental Figure S5.

2. In addition to a large-scale analysis of a high number of systems, the authors also focus on a case study – importin alpha. While the low level of sampling per complex (20 ns) for the large-scale analysis can be justified by the high number of systems studied and the general focus on just the thermodynamics of binding, I found the detailed analysis of importin alpha not to be justified at such a low level of sampling. Having a case study that illustrates the general principles captured by the large-scale analysis is a welcome idea, but in that case the level of sampling should be on at least the microsecond level to reach the standards in the field. The authors should extend their simulations of importin alpha to this level, and reanalyze their data.

While there are studies that extract detailed information about biomolecular systems based on shorter simulations (e.g., 2 ns (Spellman 2015, PLOS ONE), 10 ns (Wang 2017, J Mol Graph Model), 20 ns (Lalitha 2016, Curr Comput Aided Drug Des)), we do agree that the studies focusing on analysis of interactions should preferably be longer. However, given that the analysis of importin alpha was not the primary goal of the submitted work, we believe that investing significant resources in performing 50 times longer simulations is beyond the scope of this publication.

Instead, importin alpha is only meant as an example of how the presented data can be used and interpreted. In addition, the already performed MD simulations took 22 - 23 h for each of the complexes using 40 cores of the VSC (Flemish Supercomputer Center), making it uncertain that the microsecond simulation would be finished by the resubmission deadline.

We have therefore clearly emphasized the following two caveats in the revised section on importin alpha: 1. that the length of the simulation should preferably be longer, and 2. that the observed interaction patterns are one-time observations, given that only one simulation was performed per system (non-modified and modified complex). In addition, milder language was used to describe the conclusions of the observed interactions.

3. The authors should provide a more extensive discussion of the potential deficiencies of the MM/GBSA method for free energy calculations and discuss what they would expect to obtain with more accurate methods.

The introduction of the MM/GBSA method into the PTMs' effects prediction pipeline was discussed in our previous work (Šoštarić et. al, 2018). In general, the accuracy of MM/GBSA falls between simpler methods, such as docking and scoring, and computationally highly intensive ones, such as alchemical perturbations, which require extensive computational sampling of unphysical intermediate states and unbound and bound states of the complex (Genheden and Ryde, 2015). We have therefore found MM/GBSA to be a good trade-off for prediction of the PTMs' effects, especially when taking into account the number of the analyzed systems, as well as the size these protein complexes can reach (the largest complex in the dataset presented in the current manuscript contains 6,386 amino acids; plot depicting sizes of complexes was added as Supplemental Figure S1 in the revised work). In our previously published study, we have tested different variations of MM/GBSA, e.g., using 100 vs. 1,000 conformational snapshots from different parts of the trajectories (last 5, 7.5, and 10 ns) in order to obtain $\Delta\Delta G_{bind}$ *values, as well as discussed details such as omission of the entropy term. In addition, we compared MM/GBSA with MM/PBSA. However, we found no significant differences in our predictions due to any of these pipeline alterations.*

The pipeline applied in the submitted manuscript for predictions of effects of PTMs on stability of binding in protein complexes was also benchmarked in Šoštarić et. al, 2018 on a set of 47 mammalian protein complexes whose stability was experimentally shown to be affected by interface-located phosphorylations. When compared to FoldX (Schymkowitz et al., 2005) and Mechismo (Betts et al., 2017) methods, FoldX was found to have the lowest accuracy that did not improve by increasing the prediction threshold, and Mechismo reached approximately 70% accuracy at the cost of coverage (sensitivity) (13 and 33%), whereas MD-MM/GBSA had a much higher coverage (Figure 4 in Šoštarić et. al, 2018). We are therefore confident that MM/GBSA is a good method for the task at hand. In order to make it more clear, we have added a more detailed discussion on the performed benchmarking in the second to last paragraph of the Introduction in the revised manuscript.

Minor comments

1. L29. "Stressful" should be changed to "stress"

The correction has been made in the revised manuscript.

2. L453 should be "due to their large sizes"

The correction has been made in the revised manuscript.

3. How were the 100 conformational shapshots from the final 10 ns of each MD trajectory that were used for free energy calculation chosen? At random or from equally spaced time points? This should be mentioned in the methods.

We thank the reviewer for this remark. The conformational snapshots have been taken from equally spaced time points from the last 10 ns of the trajectory, and this is now explicitly stated in the "Binding energy calculation" section of the Methods in the revised manuscript.

4. The authors should more exhaustively cite the available literature especially on MD simulations of PTMs and their impact on fundamental biological processes (e.g. Petrov et al. 2013, PLOS Computational Biology, e1003154; Paterlini, M. G. et al. Biophys. J. 2005, 88, 3243-3251; Polyansky AA et al, J Phys Chem Lett 3: 973–976, 2012.)

We thank review for this suggestion. The available literature has been cited more thoroughly in the new version of the manuscript, particularly in the first part of the Discussion section.

Reviewer #2

In this manuscript, the authors study the effect of acetylation and phosphorylation on the stability of protein complexes. Whereas acetylation appears to have a stabilizing effect, phosphorylation seems to destabilize interfaces. The impact of these post- translational modifications (PTMs) on the conformation of complexes was also studied by calculating the root-mean-square deviation of the complexes. These results were obtained by performing molecular dynamics simulations and free energy calculations on a filtered dataset and comparing trajectories of modified versus non-modified PTM systems.

General

Overall I think the manuscript shows interesting effects of the presence of PTMs and how multiple PTMs in one system can have a very different effect than individual PTMs in the same system. My main concern is the setup of the molecular dynamics simulations. Especially, since the padding of the systems was 10 Å of water molecules and the cutoff for non-bonded interactions was 12Å for the production phase. Because compression of the initial systems as well as conformational changes of the studied proteins is common in MD, there is a possibility that the simulated proteins reach a distance shorter than 12Å between the original protein in the unit cell and one of its periodic images. This should be carefully checked and if this artifact is found in one of the systems, these simulations should be discarded and rerun as this can affect the stability and conformation of the studied complexes. Another potential issue with the simulations could be the use of a Berendsen type thermostat for the production phase instead of a Nose-Hoover thermostat and the length of the produced trajectories.

Major

Section Post-translational modifications of yeast proteins.
From line 143 to line 155, percentages of PTMs are described for different conditions. For reader clarity, would it be possible to translate these percentages into pie charts, for example, so the reader can get an overview of the discussed data in a figure as well?

We thank the reviewer for the suggestion. We have prepared 5 corresponding plots: one per PTM type (Lys acetylation, Ser phosphorylation, Thr phosphorylation, Tyr phosphorylation), as well as a plot showing all PTMs together. Each of these contains information on both normal and stress conditions, so they are easy to compare. The corresponding figure has been added in the Supplemental Information as Supplemental Figure S2.

Section Protein conformational changes due to PTMs.
I think it would be good to also show the plain RMSDs for modified and non-modified systems in a supp figure to show that the RMSDs have converged in the production phase. The backbone RMSD will show the clear change in conformation. The RMSD of the entire protein with side chains can cloud the difference in conformations.

We have prepared the corresponding multi-panel figure showing backbone RMSDs for all systems and added it to the Supplemental Information as Supplemental Figure S6. It can be seen that the large majority of the systems have indeed converged in the production phase, with a few exceptions mentioned in the figure legend, as well as in the last paragraph of the "Protein conformational changes due to PTMs" section.

Section A case study – importin alpha.
I think it would be helpful for clarity if the Importin mechanism of dimerization and activation could be illustrated with a figure in the supporting information for the first paragraph of this section.

We thank the reviewer for the suggestion. We have prepared a figure depicting the previously proposed mechanisms of importin alpha auto-inhibiton, as well as importin beta's role in its release. The figure has been added to the Supplemental Information (Supplemental Figure S11).

Section Molecular dynamics simulations.
I would strongly advice to change the order of the simulation details. Especially the equilibration and production protocol descriptions are challenging to follow.

Line 475. It would be advantageous to repeat the description of the full protocol of protein structure preparation and MD used in this manuscript as well because a large part of the findings are based on MD data.

We thank the reviewer for this remark. Because the MD-MM/GBSA pipeline used in this work has indeed previously been introduced and described in detail (Šoštarić et al. 2018), we aimed to just briefly outline it in the present work. However, we understand that it is not clear enough in the present form, and have expanded the sections on MD and MM/GBSA in the revised manuscript.

Line 487. Besides neutralizing the systems, was 150mM of salt also added to the systems?

The mentioned 150 mM of salt was used only in the MM/GBSA calculation, as mentioned in the Methods section.

Line 487, 494, 502. Cutoff for non-bonded interactions seems quite long (15Å equilibration, 12Å production) with respect to the water padding of 10 Å that was used. A major concern would be the protein in the unit cell "feeling" its periodic image. Authors should check for all trajectories if the original proteins and their periodic images are further apart than 12Å in the production runs. Some systems show large RMSDs, this could be caused by this artifact. It would be essential to check this and if the case, discard all systems with this periodic image issue.

The unit cell was built by adding the water molecules spanning 10 Å from protein in each direction, making the minimal distance between proteins in the unit cell and its periodic image 20Å. We believe that the brevity of the MD-MM/GBSA explanation in the Methods section rendered this unclear, so we added a more precise explanation in the revised manuscript.

Regarding the large values of the RMSD differences between non-modified and post-translationally modified versions of the protein complex found for some systems, the examination of these outliers (e.g., the 1VLU system) showed that they appear due to major differences between the two examined states of the complex. For example, in the mentioned system, the dissociation of protein subunits occurs only for the modified and not for the non-modified complex, while the RMSD is measured for the complex as a whole, as mentioned in the last paragraph of the "Protein conformational changes due to PTMs" section of the Results.

Line 498. Why is there a switch from amber to gromacs? 19.5 ns of production is on the short side for letting the system converge and being able to check protein stability. Would it be possible to extend the simulations to 50 ns? Were replicates with different initial velocities run to verify the reproducibility of the MD simulations?

The switch to Gromacs was done solely to save the computational resources - because its parallelization across multiple nodes works much better then Amber's at the available infrastructure (Flemish Supercomputing Center, VSC), this switch saves both resources and days of computational time. However, when working on our previous publication on this topic (MD-MMGBSA; Šoštarić et al. 2018), we found that some of the Gromacs' functionalities give peculiar results (e.g., g_mmpbsa), so we opted to perform binding energy calculation with Amber's MMGBSA.py.MPI. The resulting pipeline therefore combines the two suites, using ParmEd for switching between their respective formats.

The current length of the simulation is in agreement with the pipeline we have previously benchmarked in Šoštarić et al. 2018, where we also discussed different lengths of the MD production phase. Briefly, in our previous work, we have compared results obtained by MM/GBSA and MM/PBSA using 100 vs. 1,000 conformational snapshots taken from the last 5, 7.5 or 10 ns of the trajectory, and found no significant differences. We have further benchmarked our MD-MM/GBSA method against FoldX (Schymkowitz et al. 2005) and Mechismo (Betts et al. 2017) using a dataset of 47 mammalian complexes for which it was experimentally shown that their interface-located phosphorylations cause either stabilization (24 complexes) or destabilization (23 complexes). Benchmarking showed that MD-MM/GBSA outperforms the other two methods (Figure 4 and Supplemental table S4.2 in the previous work), using 19.5 ns for conformational sampling. Given that we are also working with large multimeric complexes (the largest complex for which we made predictions in our previous work was 6,996 residues large, and in the present work 6,386 residues). Thus, while the relative shortness of the MD simulation reflects the reasonable limitation of the resources, we believe that the conclusions would not be significantly different with somewhat longer simulation time, especially as the described benchmarking shows that the used length of MD does not prevent MD-MM/GBSA from making good predictions of PTMs' effects.

Lastly, the replicates with different initial velocities were not run to verify the reproducibility of the simulations in this work. This was, however, done within our previous work.

Line 500. The modified Berendsen thermostat is used. is this the V-rescale thermostat? Is there a reason why a Nose-Hoover thermostat was not used during production? According to my experience, Nose-Hoover is recommended as thermostat for production runs, V-rescale is usually used for the equilibration phase.

Because the described set-up was also used in the successful benchmarking of the pipeline within our previous work, we did not want to change it for predictions on the current dataset.

Line 501. Was an isotropic barostat used as all simulated systems were solvated?

Yes, Parinello-Rahman barostat was used, as mentioned in the Methods section in the revised manuscript.

Minor

Line 106. “that a significant proportions of proteome carries”. Proportions should be changed to proportion.

The correction has been made in the revised manuscript.

Line 109. “over-performs”, do the authors mean outperforms?

Indeed, that is what was meant. The correction has been made in the revised manuscript.

Lines 157-159. This sentence is a bit on the long side. Could this sentence be restructured?

The mentioned sentence has been restructured in the revised manuscript.

Line 210. “PBD” should be changed to PDB.

The correction has been made in the revised manuscript.

Line 261-261. Can the authors also show the same trend for PTMs when you only take into account residues that are located at the protein-protein interface?

The interface-located PTMs can be identified on the corresponding plot as those with non-zero contributions (previously Supplemental Figure 5, now Supplemental Figure 10). Even after excluding the sites outside of protein-protein interfaces (i.e., the data points lying on the x-axis), the remaining data points are still very much scattered, so the statement from line 261 also applies to the interface-located subset of PTMs. We have emphasized this in the mentioned section of the revised manuscript.

Line 348-349. Are the serines more often located at protein-protein interfaces than the threonines? Could this be a reason why serines have more impact on the stability than threonines?

While there are more serine than threonine phosphorylation sites both in the entire dataset, as well as in the interface-located subset of PTMs, the mentioned line refers to findings presented in Figure 2, which already shows the data for interface-located PTMs only (as PTM sites outside of interfaces have zero local contributions to binding).

Section Selection of protein structures.
In the dataset, were only dimers considered or also larger complexes? It would be nice to know the distribution.

Although the majority of complexes are dimers, our dataset also includes a number of multimeric complexes, the largest one consisting of 28 subunits. A figure depicting this distribution has been

added to the Supplemental Information (Supplemental Figure S1), alongside a plot showing the distribution of the sizes (in terms of the number of residues) for all protein systems.

Figure 2. In order to increase the readability of the figure, maybe make clear in the figure which graphs concern phosphorylation and which ones acetylation. Using the same range for the y-axes would also make it easier to compare the different PTMs. It could also be useful to put the numbers of the means in the graph for B and maybe also for A. It will make it easier for the reader to compare results.

We thank the reviewer for the valuable suggestions. All the suggested improvements were implemented into Figure 2: the violin plots corresponding to acetylation and phosphorylation were given different colors, making them visually distinguishable; the ranges used on y-axes were made uniform among all plots; the numeric values of the means have also been added. Due to these modifications, we also found it more practical to incorporate the data previously shown in panel B to the panel A, so a revised version of the figure contains a single panel.

Figure 4. It could have something to do with the PDF, but which plot is stress and which plot is normal conditions? Would it be helpful to print the means here as well?

We thank he reviewer for this remark. The conditions (normal on the left, stress on the right) were not shown due to a mistake, which has now been corrected. In addition, we also printed the means on the plots.

Figure 5. How many PTMs were considered in total? How many sequences were taken into account per bar in the histogram, are the results normalized somehow per PTM? I am just wondering how one can compare the different bars in the histogram. To me it is unclear what these bars are composed of.

When making Figure 5, all unique PTM sites analyzed in this work have been taken into account - acetylation and phosphorylation sites together independent of the conditions (normal or stress). Some sites appeared multiple times in our dataset (e.g., if a specific protein chain modified by a given PTM appeared in more than one complex; if the same PTM was identified in both normal and stress conditions; etc.), so such sites were taken into account only once for the purposes of Figure 5 in order to remove the redundancy. Hence, the total number of PTMs in the graph was 1,575. As we understand the nature of the data behind Figure 5 was not clear, we have added an extended explanation in the respective figure legend.

Figure 6. In the modified structures, I am assuming Lys463 and Ser163 are not acetylated and phosphorylated, respectively? If these residues are not modified, which nearest residues are modified and can the orientation with respect to the highlighted site be shown as well?

Indeed, Lys463 and Ser163 were not modification sites in importin alpha. In both cases, the closest PTM is located approximately 17 - 20 Å further in space. Because of their orientation and distance, it is very impractical to show these PTMs in the Figure 6 - both the un-zooming and

rotations needed to include PTMs in the figure make it difficult to see the changes in the binding on which we aimed to focus with this figure. We have therefore opted to leave this figure as is.

Supp Figure 1. It is a bit challenging to read. Could adding an additional representation help? For example, by clustering the runs by specific range in ΔΔG difference?

We have clustered the presented data based on the ΔΔG differences between normal and stress conditions, splitting the figure into 4 panels, corresponding to differences of up to 10 kcal/mol, between 10 and 20 kcal/mol, between 20 and 50 kcal/mol, and above 50 kcal/mol. We agree that making multiple panels has indeed made the figure (which is now shown as Supplemental Figure S4) easier to read.

Supp Figure 3. Maybe also show the difference between A and B so it becomes clear very quickly what the change in the distribution of secondary structure elements is for the modified residues.

We thank the reviewer for this suggestion. We have added the suggested plot as panel C in this Supplemental Figure (S8 in the revised work).

Reviewer #3

This study addresses the field of PTMs and whether they stabilize or destabilize protein interactions. Not many computational studies have attempted this. The authors have previously (in [24]) studied 3 protein complexes with the same GB/SA protocol and validated their findings against their own experimental data. Here, they now inspected 174 protein complexes having 2832 lysine acetylation and 289 phosphorylation PTMs. All of these systems were subjected to the same MD protocol, which is quite impressive. Much of this work has been conducted quite carefully, e.g. I liked their preparation of the data set and the initial statistics of PTMs.

However, I am a bit sceptical about the reported deltaG numbers.

(1) Fig. 2 suggests that all phosphorylation-PTMs are destabilizing. I don't think this is generally true. Why would then e.g. 14-3-3 domains always preferably bind to phosphorylated binding partners? The unphosphorylated variants should always have more favorable binding energies, which is not the case. I think the authors should critically reflect on whether they want to stick to their strict interpretation. At least, the authors need to find a way to convince the reader that GB/SA works fine for phosphorylation PTMs.

When it comes to local contributions, our results in fact show a mix of stabilizing and destabilizing effects for Ser/Thr/Tyr phosphorylations. This can be seen in the results presented in the Supplemental Table S2. As we realize that this is perhaps not clear from the violin plot depicted in Figure 2, we have added bar graphs showing the detailed count of individual PTM site types found to be stabilizing vs. destabilizing (both for all PTM sites and for the interface-located ones) as Supplemental Figure S3 in the revised work.

In addition, we would like to emphasize that the local effects of individual PTMs do not necessarily reflect on the global scale, as indicated in the "Co-occurring PTMs together affect protein interactions". We also added a remark on that at the end of the first paragraph in the "Acetylation and phosphorylation have different local contributions to binding" in the revised manuscript. For example, even with the local effect of phosphorylated residues on binding of the subunits being destabilizing, the overall effect on protein-protein binding can be stabilizing due to more complex effects, such as PTM sites crosstalk and induced conformational changes. We see such cases both in the data presented in this work (Supplemental Table S2), as well as in the benchmarking dataset from our previous work (Šoštarić et al. 2018, Supplemental Table S4.2; also mentioned in the Benchmarking section of the Results in our previous work).

(2) One potential source of error could in fact be the method used to compute delta-delta-G values (GB/SA). Such calculations are very popular, yet this does not mean that their results to estimate PTM energies can simply be trusted.

In https://onlinelibrary.wiley.com/doi/full/10.1002/wcms.1448 the authors reviewed the ability of various computational approaches to compute protein protein binding affinities. In section 4.1, they comment on the abilities of the GB/SA method. Based on this, the method seems to work generally acceptable, at least as a scoring method.

However, there are also other reports. E.g. https://www.ncbi.nlm.nih.gov/pmc/articles/PMC6453258/ recently studied the ability of FEP calculations and mm-GB/SA calculations to predict the effect of charge-changing sequence mutations at protein-protein interfaces. Whereas FEP worked quite well (RMSE 1.23 kcal/mol for exposed positions and 1.79 kcal/mol for buried positions), mm-GB/SA gave larger deviations (1.50 kcal/mol and 2.80 kcal/mol), see table 2. For

cases showing small effects (table 3), FEP gave an R2 value of 0.39, whereas gave mm-GB/SA gave practically zero correlation of 0.06! As a caveat of this study, one should add that the authors did not perform MD simulations in solvent (as was done in this manuscript), but simply performed a rotamer search while keeping the protein backbone fixed.

I am not aware if the accuracy of GB/SA calculations to predict delta_delta_G-values upon phosphorylation-PTMs has been benchmarked so far. A problem is likely the lack of suitable experimental benchmark data. Thus, for the moment, I suggest that the authors should add a cautious statement in the discussion section noting that the accuracy of GB/SA calculations for PTMs is still a bit unclear. I did look at their previous paper [24]. Yet, I do not consider Y2H screen data as quantitative data.

Thanks to the reviewer's comments, we realize that the validity of using the MD-MM/GBSA pipeline for prediction of effects of PTMs on protein-protein binding is not justified clearly enough in the present manuscript. We have accordingly added more details on the previous work and benchmarking in the second to last paragraph of the Introduction section of the revised manuscript.

In our previously published work, we have benchmarked the MD-MM/GBSA pipeline (Supplemental Table S4.2 in Šoštarić et al. 2018) which is applied in the present work. As a benchmarking dataset, we used a total of 47 mammalian protein-protein complexes for which it was previously experimentally shown that the interface-located phosphorylations cause stabilization (24 complexes) or destabilization (23 complexes) of binding; these data were available at Betts et al. 2017 and were not a part of the experiments done within our publication. Using this dataset, we compared predictions of effects of PTMs on protein binding (stabilizing vs. destabilizing) obtained by our MD-MM/GBSA pipeline with those of FoldX (Schymkowitz et al. 2005) and Mechismo (Betts et al. 2017). Notably, in Šoštarić et al. 2018 we also tested the variations of the MM/GBSA method before using it for the benchmarking (e.g., usage of different number of conformational snapshots, taking the snapshots from different parts of the trajectory; we also compared it with MM/PBSA).

The benchmarking results showed that FoldX has the lowest accuracy which does not improve by increasing the prediction threshold. Mechismo could reach up to 75% accuracy at the cost of coverage (sensitivity) (13 and 33%) (Figure 4 in Šoštarić et al. 2018), while our MD-MM/GBSA method has a much higher coverage. While FoldX and Mechismo made similar or slightly better predictions than MD-MM/GBSA in predicting the stabilizing effects, MD-MM/GBSA outperformed them in predicting the destabilizing ones. Another difference was in the number of predictions above the threshold considered to indicate a significant effect on binding for each method, where the numbers were quite low for FoldX and Mechismo, especially for the destabilizing effects. In addition, it should be noted that global, and not local, effects on binding were taken into account when making the predictions with MD-MM/GBSA, where the sign (negative or positive) of the $\Delta\Delta G_{bind}$ value was the main indicator of the effect.

After benchmarking, the MD-MM/GBSA pipeline was applied to predict the effects of interface-located PTMs in three multimeric complexes in Šoštarić et al. 2018. Yeast 2-hybrid method was then used to experimentally validate one of the predictions made for the exosome complex, but this was not related to the benchmarking (in which we showed that MD-MM/GBSA makes at least as good predictions of PTMs' effects as the current prediction methods using the above-mentioned benchmarking dataset, while it offers a wider spectrum of functionalities, such as taking dynamics and crosstalk into account).

(3) Only one MD simulation was performed here for each system. Hence, the conformational reorientations discussed in lines 319++ are one-time observations. It is unclear whether these are

reproducable if the simulations were repeated starting with a different random seem for assignment of velocities. Thus, a caveat should be added.

We agree with the reviewer and have pointed out this caveat in the section on importin alpha in the revised manuscript.

Further points:

(4) line 179++ the reported p-values are all extremely small, which does not fit to the overlaps seen in Fig. 2B. Maybe this results from the large number of samples? I suggest that the authors also compute Cohen's d values to estimate the effect size.

We thank the reviewer for this suggestion, which we have incorporated throughout the revised manuscript. In addition to p-values, the revised Figure 2 legend also reports Cohen's d values, which were found to range from 0.3 to 2.2 depending on the PTM sites pair.

(5) line 235: An RMSD deviation of 10 A reflects a major conformational change such as unfolding of a domain or unbinding. Stably folded domains do not show such high RMSD during 20 ns simulations.

The RMSD differences amounting to 10 Å are indeed outliers in our dataset (this can also be seen from the newly added backbone RMSDs panel, Supplemental Figure S6). As mentioned in the "Protein conformational changes due to PTMs" section of the Results, these high values occur due to major differences between non-modified and post-translationally modified complex, such as protein-protein dissociation in only one of these complexes, because the RMSD difference refers to comparison of the entire complexes. An example of such system (PDB ID: 1VLU) is mentioned in the given section of the manuscript.

(6) line 297: all values below -20 kcal/mol are clearly unrealistically low. Such extreme values are "features" of the GB/SA method, and do not exist in nature. A comment should be added.

We thank the reviewer for this suggestion. Indeed, with the MD-MM/GBSA method, the main indicator for predicting the effect of PTMs is the sign (positive vs. negative) of the $\Delta\Delta G_{bind}$ value and not the size of the value itself. We have emphasized this in the "Binding energy calculation" section in the revised manuscript.

(7) line 363++: It is well-known that a small compact folded protein would show an RMSD of 1.5 to 2 A after an MD simulation of 10 - 20 ns length.

We thank the reviewer for this remark. We have rephrased the given paragraph accordingly.

(8) line 503: PME was used for the LONG-RANGED electrostatic interactions.

The correction has been made in the revised manuscript.

Further, the manuscript would benefit from a careful grammar revision. I would e.g. add dozens of direct and indirect articles throughout the text.

We thank the reviewer for this remark. We made a thorough grammar check using the appropriate tool (Grammarly) prior to submitting the revised manuscript.
