# Supplementary material for: Molecular dynamics shows complex interplay and long-range effects of post-translational modifications in yeast protein interactions
Source: PLoS Comput Biol. 2021 May 12;17(5):e1008988. doi: 10.1371/journal.pcbi.1008988 (PMC8143416; doi:10.1371/journal.pcbi.1008988)
Supplement: Attachment — Submitted filename: Response_to_reviewers.pdf [file pcbi.1008988.s018.pdf]

Manuscript resubmission

Molecular dynamics shows complex interplay and long-range effects of post-translational modifications in yeast protein interactions

PCOMPBIOL-D-20-01903R1

Dear Dr. Dunbrack,
Dear Dr. Elofsson,

Thank you for the opportunity to resubmit our manuscript for publication as Research Article in PLOS Computational Biology. We appreciate the further constructive suggestions and believe that our manuscript has improved by the suggested edits.

Following this letter is our point-by-point response to the issues raised by reviewers, including the description of changes made in the manuscript and the supplemental material.

For the authors,

Nikolina Šoštarić

Reviewer #1

The authors have successfully addressed all of my comments.

Reviewer #2

Overall, the manuscript has improved significantly. The authors have added important information via figures. Still a few questions remain about the added figures and text.

Comments

1. Supplemental Figure S4, D: There does seem to be a difference between the normal and stress level in subfigure D compared to A,B,C. Does this have implications?

All data presented in Supplemental Figure S4 was originally a single graph. In order to make it easier to read, in the first revision of the manuscript we have split the data into four panels by clustering systems by specific range of ΔΔG difference (normal vs. stress conditions); panel D of the current Supplemental Figure S4 therefore shows systems with the largest differences (>50 kcal/mol). The ranges for panels A-D were, however, chosen arbitrarily, with the main aim of spreading out the data (more or less evenly) among the panels in order to improve readability. Because of this arbitrariness factor in choosing the ranges, we find that any potential differences between panels might be accidental rather than having meaningful implications.

2. P246, "initial structures": Just for clarification, the initial structures are the modified and non-modified structures respectively after energy minimization? So they are aligned on their respective structures, not on the same structure?

Each system was indeed aligned to its respective initial structure (i.e., non-modified to the initial non-modified, and modified to the initial modified), where "initial structure" refers to the one before energy minimization, as mentioned in P246 (P250 in the revised manuscript). This choice of reference conformations was made in order to make the data for non-modified and modified system as comparable as possible - the conformations of a non-modified and modified system before optimization are the same due to the way in which the modified system was prepared (by adding PTMs on the non-modified structure). This is also described in the Conformational changes section of the Methods.

3. Supplemental Figure 6: Thank you for adding this figure. It provides a clear view on the stability of the simulated structures. Although an additional periodic boundary analysis might not be necessary for all, could the authors please show that the structures with extreme RMSDs actually do not violate the periodic boundary conditions? This would be insightful, for example, for PDB structures: 1VLU, 2EKE, 4DL0, and 4WXA.

Indeed, given the extreme RMSD values for the mentioned four systems, it could've been expected that the violation of the periodic boundary conditions have occurred in them, so it was an oversight not to check for the minimal distance between complexes and their periodic images. We therefore thank the reviewer for this constructive observation and have made the corresponding corrections in our work.

More specifically, we have confirmed the expected PBC violation by calculating the minimal distance between proteins and their periodic images for the mentioned systems (1VLU, 2EKE, 4DL0, and 4WXA) using gmx mindist with -pi flag. Because we believe that it's still worthwhile mentioning the effects that took place in these systems, they are not entirely excluded from the revised version of the manuscript. Instead, it's heavily emphasized that the events in

these systems are exceptional (P173-177 and P256-262) and they have been removed from all analyses that include binding energy calculations, as well as from the statistics on RMSD values. The following parts of the work have been revised accordingly: Figs 2, 3, and 4; Supplemental Figs 3, 4, 5, 7, and 10; first three sheets of Supplemental Table S2; all the affected numerical values in the Results and Discussion sections (e.g., certain p-values and Cohen's d-values; notably, these changes were minor and have not affected any of the conclusions).

4. P265, "Thus, we find that the protein complexes during MD typically do acquire conformations rather distant from the initial one, with modified complexes more frequently showing larger changes than their non-modified counterparts." This finding does not seem surprising as the starting structures all originate from a non-modified form of the protein and, therefore, it can be expected that perturbation of the initial system via modifications can lead to larger deviations from the original structure compared to the unmodified protein system. Is this what the authors are trying to point out?

That was indeed the intended take-away message. We tried to make it more clear by adding "…, as might be expected given that all the initial conformations originate from non-modified versions of protein complexes." (P277-278 in the revised manuscript).

5. P274-275, "Notably, there are also a few outliers, such as the 1VLU system with RMSD value for cluster representatives larger than 20 Å." Similar to comment 3, can the individual monomers interact with their respective periodic images in 1VLU? 20 Å seems to be a large distance when taking into account that the distance between periodic images is 20 Å in the setup of the simulation box.

In agreement with the changes described under comment 3, i.e., exclusion of 1VLU from analyses, this sentence was removed from the revised manuscript.

6. P553: "using a Python script and the PyTMs". Is this python script also being provided by the authors in the supplementary information? It could potentially be advantageous for the scientific community to publish the manuscript with the python script that adds PTMs to a protein system in an automated fashion.

We thank the reviewer for this comment and fully agree that publishing this script would be advantageous for community. We have therefore added it to the Zenodo repository accompanying this work (DOI: 10.5281/zenodo.4099098) together with a small runnable example under the name PTMs_map.zip, and have added this information in the Methods section (P553) of the revised manuscript.

7. The individual simulations of the protein systems are still on the short side and have only been performed once, but if both of these properties are pointed out in the manuscript, the reader can take this into consideration.

We agree with the reviewer's comment. In addition to the description of simulations in the Methods section, these properties are also emphasized in the Results section (P380-382).

Reviewer #3

The authors have appropriately addressed my points.
